# Efficacy of glucagon-like peptide-1 receptor agonists for prevention of stroke among patients with and without diabetes: A meta-analysis with the SELECT and FLOW trails

**DOI:** 10.1016/j.ijcha.2025.101638

**Published:** 2025-03-16

**Authors:** Asmita Gera, Fakhar Latif, Vamsikalyan Borra, Sidra Naz, Vivek Mittal, Fathima Shehnaz Ayoobkhan, Tushar Kumar, Zarghoona Wajid, Novonil Deb, Tanisha Prasad, Jishanth Mattumpuram, Vikash Jaiswal

**Affiliations:** a Department of Internal Medicine, Tianjin Medical University, Wuqing District, Tianjin 301700, China; b Department of Medicine, Dow University of Health Sciences, Karachi, Pakistan; c Department of Internal Medicine, The University of Texas Rio Grande Valley, Edinburg, Texas, USA; d The University of Texas, MD Anderson Cancer Center, Texas, USA; e Department of Internal Medicine, Trinity Health Oakland/Wayne State University, MI, USA; f Department of Cardiothoracic and Abdominal Radiology, University of Washington, Seattle, Washington, USA; g Hennepin Healthcare/University of Minnesota, S8, Minneapolis, MN 55415, USA; h Department of Medicine, North Bengal Medical College, West Bengal, India; i Department of Medicine, Royal College of Surgeons, Dublin, Ireland; j Division of Cardiology, University of Louisville School of Medicine, KY 40202, United States; k Department of Cardiovascular Research, Larkin Community Hospital, South Miami, FL, USA

**Keywords:** GLP-1 RA, Diabetes, Stroke, Meta-analysis, Outcomes

## Abstract

**Background:**

Glucagon-like peptide-1 receptor agonists (GLP-1 RAs) have shown a reduction in major adverse cardiovascular events (MACE) among patients with type 2 diabetes mellitus (T2DM). However, its efficacy on cerebrovascular events is yet to be well established among diabetic and non diabetic patients.

**Objective:**

We sought to evaluate the efficacy of GLP-1 RAs on stroke risk among its different types in patients with and without Diabetes.

**Methods:**

We performed a systematic literature search on PubMed, EMBASE, and ClinicalTrials.gov for relevant randomized controlled trials (RCTs) from inspection until 15th July 2024, without any language restrictions. Odds ratios (OR) and 95 % confidence intervals (CI) were pooled using a random-effect model, and a p-value of < 0.05 was considered statistically significant.

**Results:**

A total of 11 RCTs with 85,373 patients were included (43,339 in GLP-1 RA and 42,034 in the placebo group) in the analysis. The mean age of the patients in GLP-1 RAs and the placebo groups was 63.5 and 63.1 years, respectively. Pooled analysis of primary and secondary endpoints showed that GLP-1 RAs significantly reduced the risk of incidence of stroke by 15 % (OR, 0.85(95 %CI: 0.77–0.93), *P* < 0.001) and nonfatal stroke by 13 % (OR, 0.87(95 %CI: 0.79–0.95), *P* < 0.001) compared with placebo. However, the risk of fatal stroke (OR, 0.94(95 %CI: 0.75–1.17), *P =* 0.56) was comparable between both groups of patients. Similarly, the risk of serious adverse events such as cerebrovascular accident (OR, 0.75(95 %CI: 0.57–1.00), *P =* 0.05), hemorrhagic stroke (OR, 0.82(95 %CI: 0.42–1.60), *P =* 0.57, and ischemic stroke (OR, 0.85(95 %CI: 0.64–1.13), *P =* 0.26) was comparable between GLP-1RAs and placebo.

**Conclusion:**

Treatment with GLP-1 receptor agonists has beneficial effects in reducing the risk of stroke, and nonfatal stroke in patients with and without diabetes. However, no such effect was observed for fatal stroke.

## Highlights

• GLP-1 receptor agonists have shown cardioprotective effects in previous *meta*-analyses among diabetic patients.
• This present meta analysis has the novelty of including most recently published randomized trials such as FLOW, and SELECT trials and patients with a wide spectrum of disease evaluating risk of stroke and adverse events related to stroke.
• GLP-1 receptor agonists has beneficial effects in reducing the risk of stroke, and nonfatal stroke in patients with and without diabetes.
• Adverse events related to stroke were comparable, and risk of cerebrovascular accident was lower among GLP-1 receptor agonists group of patients.

## Graphical abstract

**Figure:**
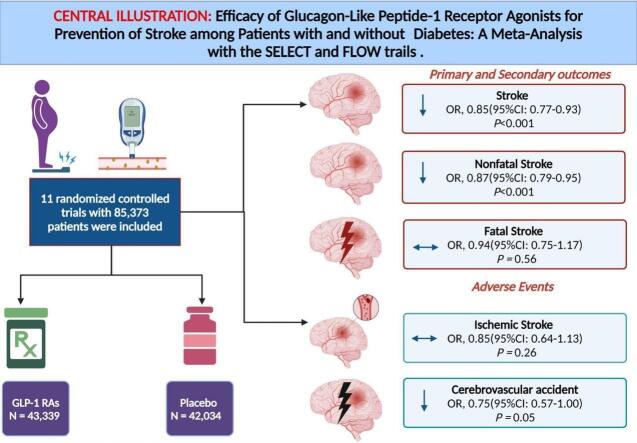

## 1 Introduction

Type 2 diabetes mellitus (T2DM) is one of the chronic and rapidly growing metabolic disorders worldwide [1]. Long-standing diabetes mellitus leads to various complications, including microvascular and macrovascular complications [2]. A *meta*-analysis of 102 prospective studies showed that patients with DM are at a two-fold risk of coronary heart disease, stroke, and death compared to other risk factors [2]. Tight glycemic control has been shown to reduce microvascular complications, but controversial results have been seen for macrovascular complications [3], [4]. However, various studies indicate that some hypoglycemic medications, despite being effective in lowering blood glucose, may increase the risk of cardiovascular events [5], [6]. Due to these findings, since 2008, cardiovascular outcomes data has been required from randomized controlled trials (RCTs) for granting new anti-diabetic medications, including Glucagon-like peptide-1 (GLP-1) analogs [7], [8].

GLP-1 analogs are glucose-lowering medications for managing various metabolic diseases, including type 2 diabetes mellitus (T2DM), overweight, and obesity [9]. They act on GLP-1 receptors in multiple organ systems, including the central nervous system, heart, kidneys, and pancreas, and food is the strongest stimulus for their secretion [10]. Various GLP-1 analog medications have been approved so far for managing metabolic diseases. With RCTs requiring cardiovascular outcome data for granting new diabetic medications, GLP-1 analogs were shown to have reduced major adverse cardiovascular events (MACE) in T2DM patients [11], [12].

However, their efficacy in reducing cerebrovascular events has not been well-established in diabetic and non diabetic patients, with controversial results [13], [14], [15]. Due to this, we have conducted a *meta*-analysis on stroke outcomes by pooling data from RCTs to evaluate the efficacy of GLP-1 RAs on stroke risk in patients with and without diabetes.

## 2 Methods

This *meta*-analysis was conducted and reported following the PRISMA (Preferred reporting items for systematic review and Meta-analysis) 2020 guidelines [16] and performed according to established methods, as described previously [17], [18], [19].

### 2.1 Search strategy

We conducted a systematic literature search in PubMed, Embase, and ClinicalTrial.gov using predefined MESH terms by using “AND” and “OR”. The following search terms were used: (((((((((((((((((((diabetes mellitus[MeSH Terms]) OR (type 2 diabetes mellitus[MeSH Terms])) AND (glucagon like peptide1[MeSH Terms])) OR (glucagon-like-peptide-1 receptor agonist[Other Term])) OR (semaglutide[Other Term])) OR (liraglutide[Other Term])) OR (albiglutide[Other Term])) OR (exenatide[Other Term])) OR (tirzepatide[Other Term])) AND (type 2 diabetes mellitus[MeSH Terms]))) AND (obesity [MeSH Terms]))) AND (acute stroke[MeSH Terms])) OR (stroke[Other Term])) OR (ischemic stroke[MeSH Terms])) OR (hemorrhagic stroke[MeSH Terms])) AND (fatal outcomes[MeSH Terms]). We queried databases from their search inception up until 15th July 2024 without any restrictions on the language of the studies.

All the studies were carefully screened and exported to the Mendeley reference manager, which handles searched citations. A manual check was carried out to cross-check for any remaining duplicates. Two reviewers (V.M and F. S. A) reviewed the papers based on the title and abstract. Another author (A.G) arbitrated discrepancies regarding the inclusion of studies.

### 2.2 Eligibility criteria

We included only clinical trials involving patients ≥ 18 years of age with and without diabetes, and obesity. It was decided to include studies with two arms in one, with GLP-1RAs used as an intervention and placebo as a control group. Studies must have reported outcomes of interest (primary and secondary outcomes). Studies performed on animals, reviews, case reports, case series, studies with a single arm or without GLP-1 RA as an intervention, or studies without outcomes of interest were excluded from the review.

### 2.3 Clinical outcomes

The primary outcome of this *meta*-analysis was stroke. Secondary outcomes include fatal stroke, nonfatal stroke, and adverse events such as ischemic stroke, hemorrhagic stroke, embolic stroke, and cerebrovascular accident.

### 2.4 Data extraction and quality assessment

Data from the eligible studies, such as demographic, study design, comorbidity, follow-up, and outcomes between GLP-1 RA and placebo groups, were extracted to a Microsoft Excel® 2019 spreadsheet by two authors (T.K and S.N).

Two authors (F.S.A and N.D) independently assessed the quality of the included studies using Cochrane Collaboration’s tool for assessing the risk of bias in randomized controlled trials.^x^

### 2.5 Statistical analysis

Baseline continuous variables were summarized in mean (standard deviation), whereas dichotomous variables were described in frequency or percentage. We performed a conventional *meta*-analysis for primary and secondary outcomes and adopted the DerSimonian and Laird random-effect model for the study variations. Outcomes were reported as pooled odds ratio (OR) and their corresponding 95 % confidence interval (95 % CI). Statistical significance was met if the 95 % CI did not cross the numeric “1″ and the two-tailed p-value was less than 0.05. We considered a two-tailed p-value of less than 0.05 to be statistically significant. In addition, we assessed the between-study heterogeneity using the Higgins I-square (I^2^) test, with I^2^ values < 75 % considered mild-moderate and > 75 % considered high [20]. All statistical work, inclusive analysis, and graphical illustrations were conducted using STATA (version 17.0, StataCorp) [21].

## 3 Results

**Fig. 1:**
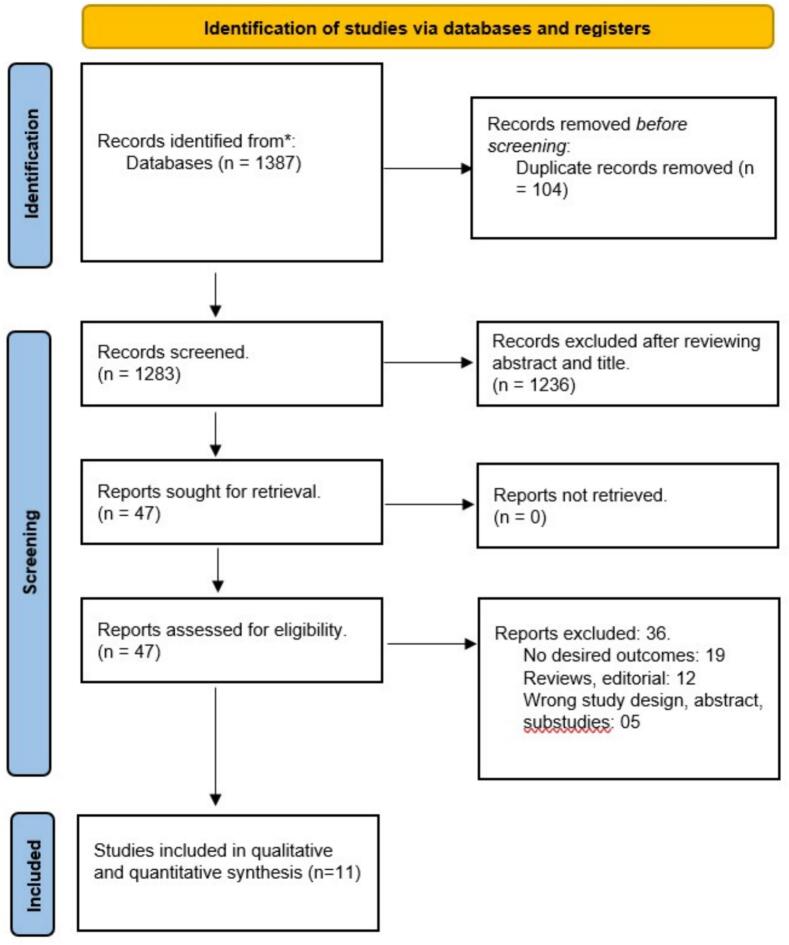
Prisma flow diagram.

The preliminary database search using the pre-specified keywords yielded 1387 articles, of which 104 duplicate studies were excluded. 1236 studies were further excluded from the initial post-title and abstract screening based on the inclusion and exclusion criteria and comparison arm. The full-text review was conducted for the remaining 47 articles identified during the search period, of which 36 studies were excluded: no desired outcomes, single arm, conference abstract, studies without any desired outcome. Hence, a total of 11 studies met the eligibility criteria and were included in the *meta*-analysis [11], [13], [15], [14], [22], [23], [24], [25], [26], [12], [27]. The Preferred Reporting Items for Systematic Reviews and Meta-Analyses (PRISMA) flow diagram is depicted in Fig. 1.

A total of 11 randomized controlled trials with 85,373 patients were included (43,339 in GLP-1 RA and 42,034 in the placebo group) in the analysis. The mean age of the GLP-1 RAs group was 63.5 years, and 63.1 years in the placebo group. The median follow-up duration was 2.1 years. The study characteristics, demographics, and comorbidities are presented in Table 1.

**Table 1:** Baseline characteristic of included studies arranged in form of (GLP-1 RA/Placebo).

**Trials**		**Sample Size**	**Age, Years**	**Female, n**	**GLP-1 RA**	**Diabetes mellitus**	**Heart failure**	**Prior stroke**	**Followup, years**
**FLOW, 2024**[24]	GLP1	1767	66.6	519	Semaglutide	1767	342	−	3.4
	Placebo	1766	66.7	530		1766	336	−	
**SELECT, 2023**[26]	GLP1	8803	61.6	2448	Semaglutide	0	2155	2058	3.31
	Placebo	8801	61.6	2424		0	2131	2052	
**EXSCEL, 2017**[23]	GLP1	7356	62	2794	Exenatide	7356	1161	1233	3.2
	Placebo	7396	62	2809		7396	1228	1276	
**HARMONY, 2018**[25]	GLP1	4731	64.1	1427	Albiglutide	4731	954	827	1.6
	Placebo	4732	64.2	1467		4732	968	854	
**REWIND, 2019**[13]	GLP1	4949	66.2	2306	Dulaglutide	4949	421	−	5.4
	Placebo	4952	66.2	2283		4952	432	−	
**PIONEER 6, 2019**[12]	GLP1	1591	66	507	Semaglutide	1591	188	184	1.3
	Placebo	1592	66	500		1592	200	165	
**ELIXA, 2015**[14]	GLP1	3034	59.9	923	Lixisenatide	3034	682	143	2.1
	Placebo	3034	60.6	938		3034	676	188	
**LEADER, 2016**[22]	GLP1	4668	64.2	183	Liraglutide	4668	835	730	3.8
	Placebo	4672	64.4	209		4672	832	777	
**SUSTAIN-6, 2016**[11]	GLP1	1648	64.7	304(37)	Semaglutide	1648	381	178	2.1
	Placebo	1649	64.7			1649	396	205	
**AMPLITUDE-O, 2021**[15]	GLP1	2717	64.6	925	Efpeglenatide	2717	487	−	1.81
	Placebo	1359	64.4	419		1359	250	−	
**FREEDOM CVO, 2022**[27]	GLP1	2075	63	778	Exenatide	2075	325	258	1.2
	Placebo	2081	62.7	747		2081	343	212	

### 3.1 Quality assessment

Using the ROB2 tool to assess the methodological quality of the included randomized controlled trials (RCTs), it was determined that 8 studies exhibited high methodological rigor, indicating a low risk of bias and thus ensuring their results can be regarded as robust and reliable. On the contrary, while 3 RCTs were classified as having “some concerns” about their methodological quality. (Supplementary Fig. 1**A-B**).

### 3.2 Meta-analysis of primary and secondary outcomes

**Fig. 2:**
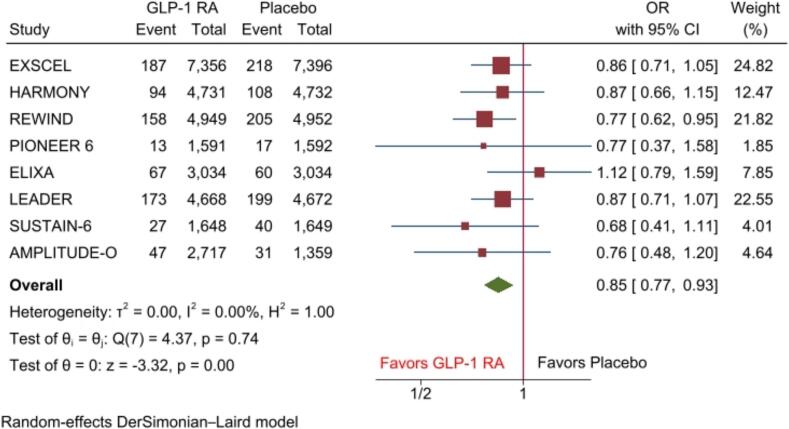
Forest plots of primary outcomes including risk of stroke.

**Fig. 3:**
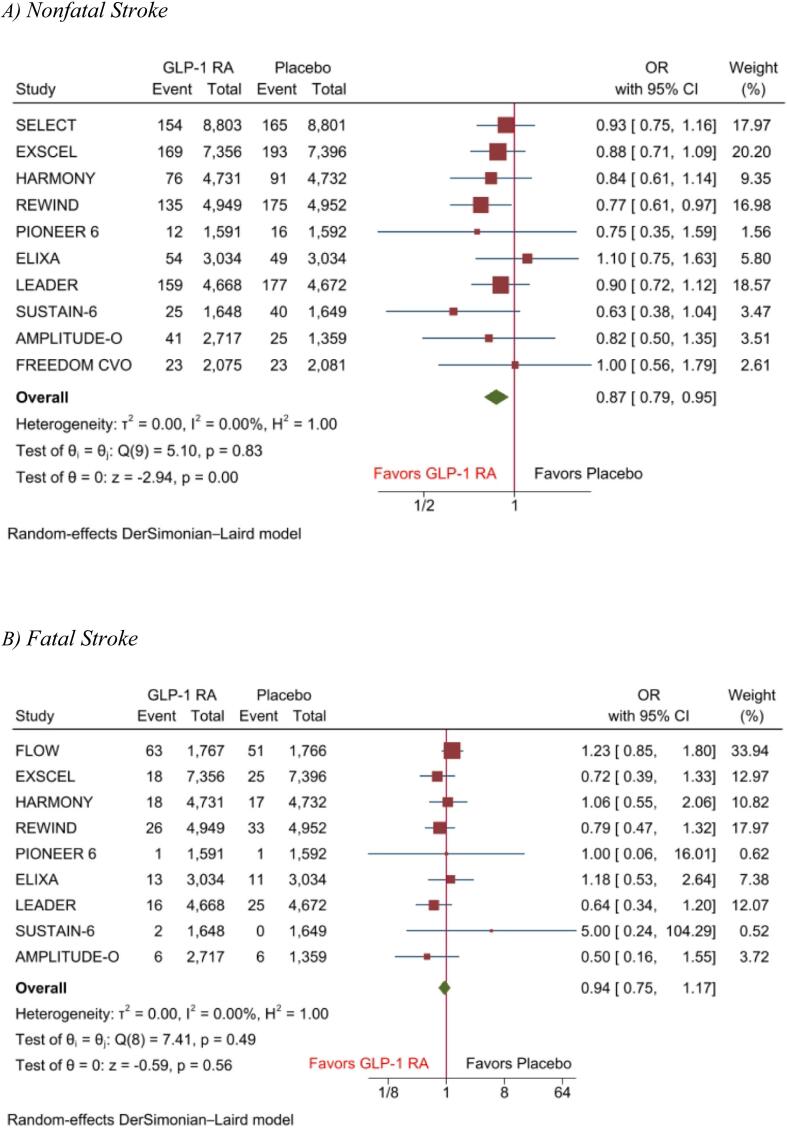
Forest plot of secondary outcome including A) Nonfatal Stroke, B) Fatal Stroke.

The pooled analysis of primary and secondary endpoints showed that GLP-1 RAs significantly reduced the risk of stroke by 15 % (OR, 0.85(95 %CI: 0.77–0.93), P < 0.001), I2 = 0) (Fig. 2), nonfatal stroke by 13 % (OR, 0.87(95 %CI: 0.79–0.95), P < 0.01), I2 = 0) when compared with placebo. However, the risk of fatal stroke was comparable between GLP-1 RA and placebo groups (OR, 0.94(95 %CI: 0.75–1.17), P = 0.56), I2 = 0) (Fig. 3**A-B**).

**Meta-analysis of adverse events**

**Fig. 4:**
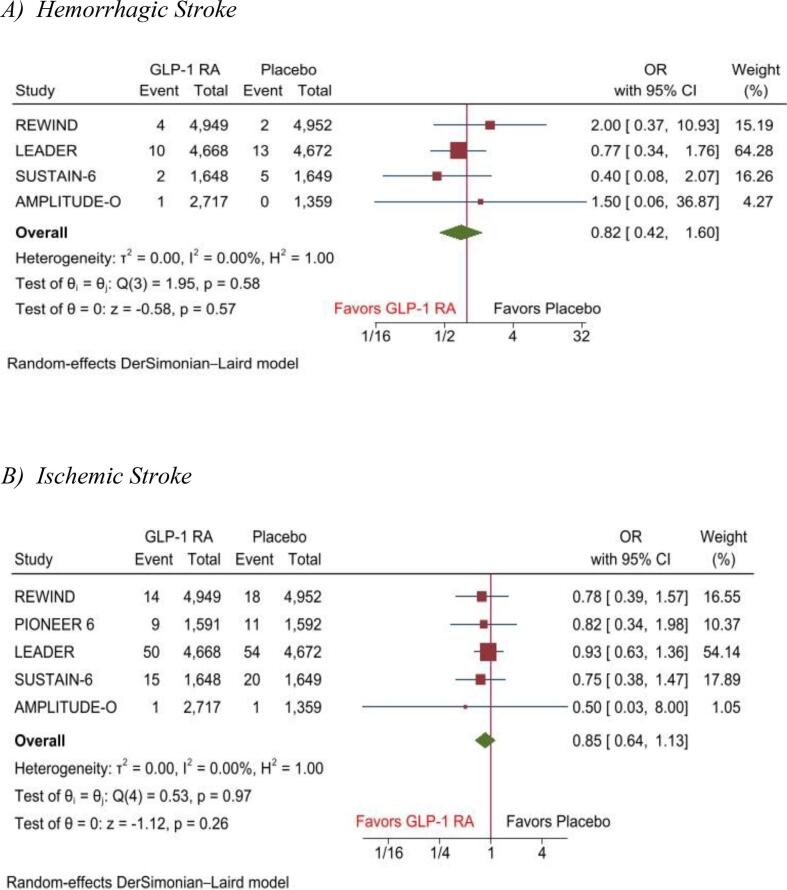

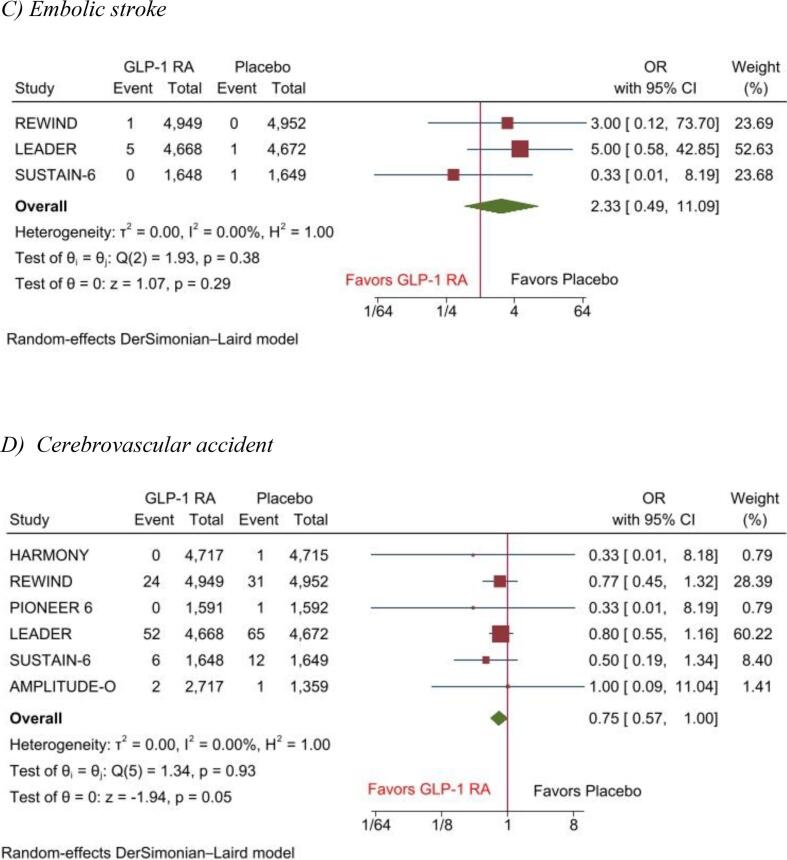
Forest plot of adverse event outcome of A) Hemorrhagic Stroke, B) Ischemic Stroke, C) Embolic stroke, D) Cerebrovascular accident. **Central Illustration:** Efficacy of Glucagon-Like Peptide-1 Receptor Agonists for Prevention of Stroke among Patients with and without Diabetes: A Meta-Analysis with the SELECT and FLOW trails.

Pooled analysis showed that the risk of hemorrhagic stroke (OR, 0.82(95 %CI: 0.42–1.60), P = 0.57), I2 = 0), ischemic stroke (OR, 0.85(95 %CI: 0.64–1.13), P = 0.26), I2 = 0), and embolic stroke (OR, 2.33(95 %CI: 0.49–11.09), P = 0.29), I2 = 0) was comparable between GLP-1 RA and placebo. However, a nonsignificant reduction in cerebrovascular accidents was observed among the GLP-1 RA group (OR, 0.75(95 %CI: 0.57–1.00), P = 0.05), I2 = 0) (Fig. 4).

**Publication bias**

Assessment of publication bias through funnel plot visualization showed that there was no evidence of publication bias for the primary outcome, and secondary outcomes. **(**Supplementary Fig. 2, Fig. 3, Fig. 4**).**

## 4 Discussion

Our comprehensive analysis, incorporating 11 RCTs with 85,373 patients, evaluated the therapeutic efficacy and safety of GLP-1 RA for stroke risk in patients with and without diabetes. When compared with placebo, GLP-1RA demonstrated a 15 % reduction in the risk of stroke. Additionally, the risk of nonfatal stroke was reduced by 13 %, while the risk of fatal stroke was comparable between both cohorts (**Central Illustration**). The risk of serious adverse events, including hemorrhagic stroke, and ischemic stroke, was also comparable. However, the risk of cerebrovascular accident found to be lower among GLP-1 receptor agonists.

The cardioprotective and neuroprotective effects of GLP-1RAs are well-established. These drugs are recognized for their capacity to mitigate TNF-α-induced oxidative stress and inflammation in endothelial cells through mechanisms involving calcium and AMPK [28], [29]. These agents effectively diminish the incidence and progression of early-stage atherosclerotic plaque formation and enhance plaque stability [30]. Moreover, GLP-1RAs are notable for their ability to lower levels of inflammatory cytokines, including TNF-α, IL-1β, and IL-6 [31]. They also exhibit antioxidant and neuroprotective effects by upregulating vascular endothelial growth factor production and decreasing proinflammatory cytokine production [32], [33]. Additionally, GLP-1RAs can stimulate the expression of several antioxidant genes downstream of protein kinase A (PKA) and cAMP response element-binding (CREB) protein, contributing meaningfully to reducing oxidative stress [34]. Furthermore, attenuation of the toxic effects of glucose by impeding the ERK1/2 and PI3K/Akt signaling pathways is also mediated by GLP-1RAs [35]. As such, intracerebroventricular administration of GLP-1RA has been shown to reduce cerebral infarct volume in rats subjected to ischemia–reperfusion injury [32], [36]. Overall, these findings suggest that GLP-1RAs possess anti-atherosclerotic and vasculoprotective properties, labeling it as the sole drug class among the new hypoglycemic agents that significantly lower the risk of stroke [37].

Our findings concluded significant benefits for cerebrovascular outcomes, which align with previously available literature. A *meta*-analysis of 7 CVOTs proposed similar results by showcasing that GLP-1RAs reduced the risk of stroke by 16 %, while the risk of fatal stroke was comparable between both cohorts [38]. Another *meta*-analysis based on 28 RCTs corroborated our findings and suggested that GLP-1RAs are associated with a 15 % risk reduction for non-fatal stroke [39]. However, it is noteworthy that this *meta*-analysis pooled only the studies catering to type 2 diabetes mellitus patients, unlike the analysis of this study. Although our analysis consistently demonstrated the neuroprotective effects of GLP-1RAs, it showed that these drugs did not lower the risk for hemorrhagic stroke with statistical significance. This finding is in line with the *meta*-analysis of Wei et al., which indicated similar results because of the low number of events and low power of their analysis [40]. Interestingly, Wei et al. also demonstrated that GLP-1RAs significantly reduce the risk of ischemic stroke, which contradicts our findings.

Our *meta*-analysis demonstrated several strengths, underscoring the robustness and reliability of our findings. To our best knowledge, this is the very first *meta*-analysis to include recently published FLOW randomized trial and evaluate the safety and efficacy of GLP-1 RA for risk of stroke and its types. Secondly, the outcomes exhibited low heterogeneity, indicating a high degree of consistency across the included studies. This low variability enhances the credibility of our conclusions, as the results are less likely to be influenced by between-study differences. Thirdly, all outcomes were reported by a substantial number of studies. This extensive reporting enhances the validity of our results by ensuring that the findings are not based on a limited or skewed dataset. Lastly, our analysis encompassed a large sample size, significantly reducing the risk of bias and increasing the statistical power of our findings. The inclusion of a vast number of participants allows for more precise estimates of the effect sizes and improves the generalizability of our results to broader populations.

Despite the robustness of our *meta*-analysis, we acknowledge several limitations of this study. Firstly, the included studies lacked specific data on the recurrence of stroke or mortality following a stroke. This absence of detailed outcome data limited our ability to fully assess the long-term effects and prognosis associated with the GLP-1RAs. Secondly, our *meta*-analysis failed to incorporate disabling stroke as an outcome because of the absence of specific data on disabling stroke outcomes across the included studies. This restricts the interpretation of GLP-1 RA efficacy in preventing strokes with long-term functional impairments. Thirdly, we did not perform subgroup analyses based on prior history of stroke or important risk factors for stroke, such as age. The failure to stratify the data according to these critical variables obscured potential variations in the effects of GLP-1RAs among different patient populations, thereby limiting the clinical applicability of our findings. Fourthly, some of our outcomes were non-significant, which may reflect either a true lack of effect or insufficient statistical power to detect the differences. This limitation suggests that further research with larger sample sizes may be needed to draw more definitive conclusions. Finally, we failed to take into account the impact of eGFR on the efficacy of GLP-1RA, which might have added value to our existing analysis.

## 5 Conclusion

This *meta*-analysis demonstrates that GLP-1RAs significantly reduce the overall risk of stroke by 15 % and nonfatal stroke by 13 %. The safety profile of GLP-1RAs is favorable, with no significant differences in serious adverse events, including cerebrovascular accident, hemorrhagic stroke, and ischemic stroke. These neuroprotective effects are likely due to reduced oxidative stress, inflammation, and atherosclerotic plaque formation. While the benefits of GLP-1RAs are evident, further research is needed to fully understand their long-term effects and impact on different patient subgroups.

Ethical approval: Not required.

**Financial support**

This analysis did not receive any specific grant from funding agencies in the public, commercial, or not-for-profit sectors.

## CRediT authorship contribution statement

**Asmita Gera:** Writing – original draft, Investigation, Conceptualization. **Fakhar Latif:** Writing – original draft. **Vamsikalyan Borra:** Writing – original draft. **Sidra Naz:** Writing – original draft, Methodology, Data curation. **Vivek Mittal:** Writing – original draft, Methodology, Data curation. **Fathima Shehnaz Ayoobkhan:** Writing – original draft, Methodology, Data curation. **Tushar Kumar:** Writing – original draft, Investigation. **Zarghoona Wajid:** Writing – original draft, Visualization, Validation, Resources, Investigation. **Novonil Deb:** Writing – review & editing, Resources, Investigation. **Tanisha Prasad:** Writing – review & editing, Writing – original draft. **Jishanth Mattumpuram:** Writing – review & editing, Writing – original draft, Visualization, Validation, Supervision. **Vikash Jaiswal:** Writing – review & editing, Writing – original draft, Visualization, Validation, Supervision, Software, Resources, Project administration, Formal analysis, Conceptualization.

## Declaration of competing interest

The authors declare the following financial interests/personal relationships which may be considered as potential competing interests: Vikash Jaiswal serve as peer reviewer for IJC Heart and Vasculature.

## References

[1] Saeedi P., Petersohn I., Salpea P. (2019). Global and regional diabetes prevalence estimates for 2019 and projections for 2030 and 2045: Results from the international diabetes federation diabetes atlas, 9th edition. DiabetesRes.Clin. Pract..

[2] The Emerging Risk Factors Collaboration (2010). Diabetes mellitus, fasting blood glucose concentration, and risk of vascular disease: a collaborative meta-analysis of 102 prospective studies. Lancet.

[3] Zoungas S., Arima H., Gerstein H.C. (2017). Effects of intensive glucose control on microvascular outcomes in patients with type 2 diabetes: a meta-analysis of individual participant data from randomised controlled trials. Lancet Diabetes Endocrinol..

[4] Turnbull F.M., Abraira C., Anderson R.J. (2009). Intensive glucose control and macrovascular outcomes in type 2 diabetes. Diabetologia.

[5] Goldfine A.B. (2008). Assessing the cardiovascular safety of diabetes therapies. N. Engl. J. Med..

[6] Lago R.M., Singh P.P., Nesto R.W. (2007). Congestive heart failure and cardiovascular death in patients with prediabetes and type 2 diabetes given thiazolidinediones: a meta-analysis of randomised clinical trials. Lancet.

[7] Menon V., Lincoff A.M. (2014). Cardiovascular safety evaluation in the development of new drugs for diabetes mellitus. Circulation.

[8] guideline-clinical-investigation-medicinal-products-treatment-or-prevention-diabetes-mellitus-revision-2_en.

[9] Michos E.D., Lopez-Jimenez F., Gulati M. (2023). Role of glucagon-like peptide-1 receptor agonists in achieving weight loss and improving cardiovascular outcomes in people with overweight and obesity. J. Am. Heart Assoc..

[10] Muzurović E.M., Volčanšek Š., Tomšić K.Z. (2022). Glucagon-like peptide-1 receptor agonists and dual glucose-dependent insulinotropic polypeptide/glucagon-like peptide-1 receptor agonists in the treatment of obesity/metabolic syndrome, prediabetes/diabetes and non-alcoholic fatty liver disease-current evidence. J. Cardiovasc. Pharmacol. Ther..

[11] Marso S.P., Bain S.C., Consoli A. (2016). Semaglutide and cardiovascular outcomes in patients with type 2 diabetes. N. Engl. J. Med..

[12] Husain M., Birkenfeld A.L., Donsmark M. (2019). Oral semaglutide and cardiovascular outcomes in patients with type 2 diabetes. N. Engl. J. Med..

[13] Gerstein H.C., Colhoun H.M., Dagenais G.R. (2019). Dulaglutide and cardiovascular outcomes in type 2 diabetes (REWIND): a double-blind, randomised placebo-controlled trial. Lancet.

[14] Pfeffer M.A., Claggett B., Diaz R. (2015). Lixisenatide in patients with type 2 diabetes and acute coronary syndrome. N. Engl. J. Med..

[15] Gerstein H.C., Sattar N., Rosenstock J. (2021). Cardiovascular and renal outcomes with efpeglenatide in type 2 diabetes. N. Engl. J. Med..

[16] Haddaway N.R., Page M.J., Pritchard C.C., McGuinness L.A. (2022). PRISMA2020: An R package and Shiny app for producing PRISMA 2020-compliant flow diagrams, with interactivity for optimised digital transparency and Open Synthesis. Campbell Syst. Rev..

[17] Jaiswal V., Hameed M., Naz S. (2023). Efficacy of lenvatinib versus sorafenib in the primary treatment of advanced hepatocellular carcinoma: A meta-analysis. JGH Open..

[18] Jaiswal V., Sawhney A., Nebuwa C. (2024). Association between testosterone replacement therapy and cardiovascular outcomes: A meta-analysis of 30 randomized controlled trials. Prog Cardiovasc Dis. Published Online April 5.

[19] Mattumpuram J., Maniya M.T., Faruqui S.K., Ahmed A., Jaiswal V., Harshakumar S.P. (2024). Cardiovascular and cerebrovascular outcomes with vitamin D supplementation: a systematic review and meta-analysis. Curr. Probl. Cardiol..

[20] Higgins J.P.T., Thompson S.G., Deeks J.J., Altman D.G. (2003). Measuring inconsistency in meta-analyses. Br. Med. J..

[21] STATA Corp. Stata Statistical Software: Release 18.

[22] Marso S.P., Daniels G.H., Brown-Frandsen K. (2016). Liraglutide and cardiovascular outcomes in type 2 diabetes. N. Engl. J. Med..

[23] Holman R.R., Bethel M.A., Mentz R.J. (2017). Effects of once-weekly exenatide on cardiovascular outcomes in type 2 diabetes. N. Engl. J. Med..

[24] Perkovic V., Tuttle K.R., Rossing P. (2024). Effects of semaglutide on chronic kidney disease in patients with type 2 diabetes. N. Engl. J. Med..

[25] Nauck M.A., Stewart M.W., Perkins C. (2016). Efficacy and safety of once-weekly GLP-1 receptor agonist albiglutide (HARMONY 2): 52 week primary endpoint results from a randomised, placebo-controlled trial in patients with type 2 diabetes mellitus inadequately controlled with diet and exercise. Diabetologia..

[26] Lincoff A.M., Brown-Frandsen K., Colhoun H.M. (2023). Semaglutide and cardiovascular outcomes in obesity without diabetes. N. Engl. J. Med..

[27] Ruff C.T., Baron M., Im K., O’Donoghue M.L., Fiedorek F.T., Sabatine M.S. (2022). Subcutaneous infusion of exenatide and cardiovascular outcomes in type 2 diabetes: a non-inferiority randomized controlled trial. Nat. Med..

[28] Hiatt W.R., Kaul S., Smith R.J. (2013). The cardiovascular safety of diabetes drugs — insights from the rosiglitazone experience. N. Engl. J. Med..

[29] N.M. Krasner, Y. Ido, N.B. Ruderman, J.M. Cacicedo, Glucagon-like peptide-1 (GLP-1) analog liraglutide inhibits endothelial cell inflammation through a calcium and AMPK dependent mechanism, Bauer PM, ed. PLoS ONE. 2014;9(5):e97554. doi:10.1371/journal.pone.0097554. 10.1371/journal.pone.0097554 PMC4023984 24835252

[30] Gaspari T., Welungoda I., Widdop R.E., Simpson R.W., Dear A.E. (2013). The GLP-1 receptor agonist liraglutide inhibits progression of vascular disease via effects on atherogenesis, plaque stability and endothelial function in an ApoE −/− mouse model. Diab. Vasc. Dis. Res...

[31] Hogan A.E., Gaoatswe G., Lynch L. (2014). Glucagon-like peptide 1 analogue therapy directly modulates innate immune-mediated inflammation in individuals with type 2 diabetes mellitus. Diabetologia..

[32] Sato K., Kameda M., Yasuhara T. (2013). Neuroprotective effects of liraglutide for stroke model of rats. Int. J. Mol. Sci..

[33] Kim S., Jeong J., Jung H.S. (2017). Anti-inflammatory effect of glucagon like peptide-1 receptor agonist, exendin-4, through modulation of IB1/JIP1 expression and JNK signaling in stroke. Experimental Neurobiology..

[34] Oeseburg H., de Boer R.A., Buikema H., van der Harst P., van Gilst W.H., Silljé H.H.W. (2010). Glucagon-Like Peptide 1 Prevents Reactive Oxygen Species–Induced Endothelial Cell Senescence Through the Activation of Protein Kinase A. Arterioscler. Thromb. Vasc. Biol...

[35] Shi L., Ji Y., Jiang X. (2015). Liraglutide attenuates high glucose-induced abnormal cell migration, proliferation, and apoptosis of vascular smooth muscle cells by activating the GLP-1 receptor, and inhibiting ERK1/2 and PI3K/Akt signaling pathways. CardioVasc. Diabetol..

[36] Lim S., Oh T.J., Dawson J., Sattar N. (2020). Diabetes drugs and stroke risk: Intensive versus conventional glucose‐lowering strategies, and implications of recent cardiovascular outcome trials. DiabetesObes. Metab..

[37] Lin D.S.H., Lee J.K., Hung C.S., Chen W.J. (2021). The efficacy and safety of novel classes of glucose-lowering drugs for cardiovascular outcomes: a network meta-analysis of randomised clinical trials. Diabetologia..

[38] Bellastella G., Maiorino M.I., Longo M. (2020). Glucagon-like peptide-1 receptor agonists and prevention of stroke systematic review of cardiovascular outcome trials with meta-analysis. Stroke.

[39] Banerjee M., Pal R., Mukhopadhyay S., Nair K. (2023). GLP-1 receptor agonists and risk of adverse cerebrovascular outcomes in type 2 diabetes: a systematic review and meta-analysis of randomized controlled trials. J. Clin. Endocrinol. Metab..

[40] Wei J., Yang B., Wang R. (2022). Risk of stroke and retinopathy during GLP-1 receptor agonist cardiovascular outcome trials: An eight RCTs meta-analysis. Front. Endocrinol..

