# Supplementary material for: Efficacy of glucagon-like peptide-1 receptor agonists for prevention of stroke among patients with and without diabetes: A meta-analysis with the SELECT and FLOW trails
Source: Int J Cardiol Heart Vasc. 2025 Mar 16;57:101638. doi: 10.1016/j.ijcha.2025.101638 (PMC11957674; doi:10.1016/j.ijcha.2025.101638)
Supplement: Supplementary Data 1 [file mmc1.docx]

**SUPPLEMENTARY FIGURES:**

**Supplementary Figure 1:** Quality assessment using Cochrane Risk of Bias tool for randomized controlled trials (RCTs) A) Risk of Bias summary, B) Risk of Bias graph.

**Supplementary Figure 2:** Funnel plot of primary outcome stroke.

**Supplementary Figure 3:** Funnel plot for non-fatal stroke.

**Supplementary Figure 4:** Funnel plot for fatal stroke.

*A) Risk of Bias summary*


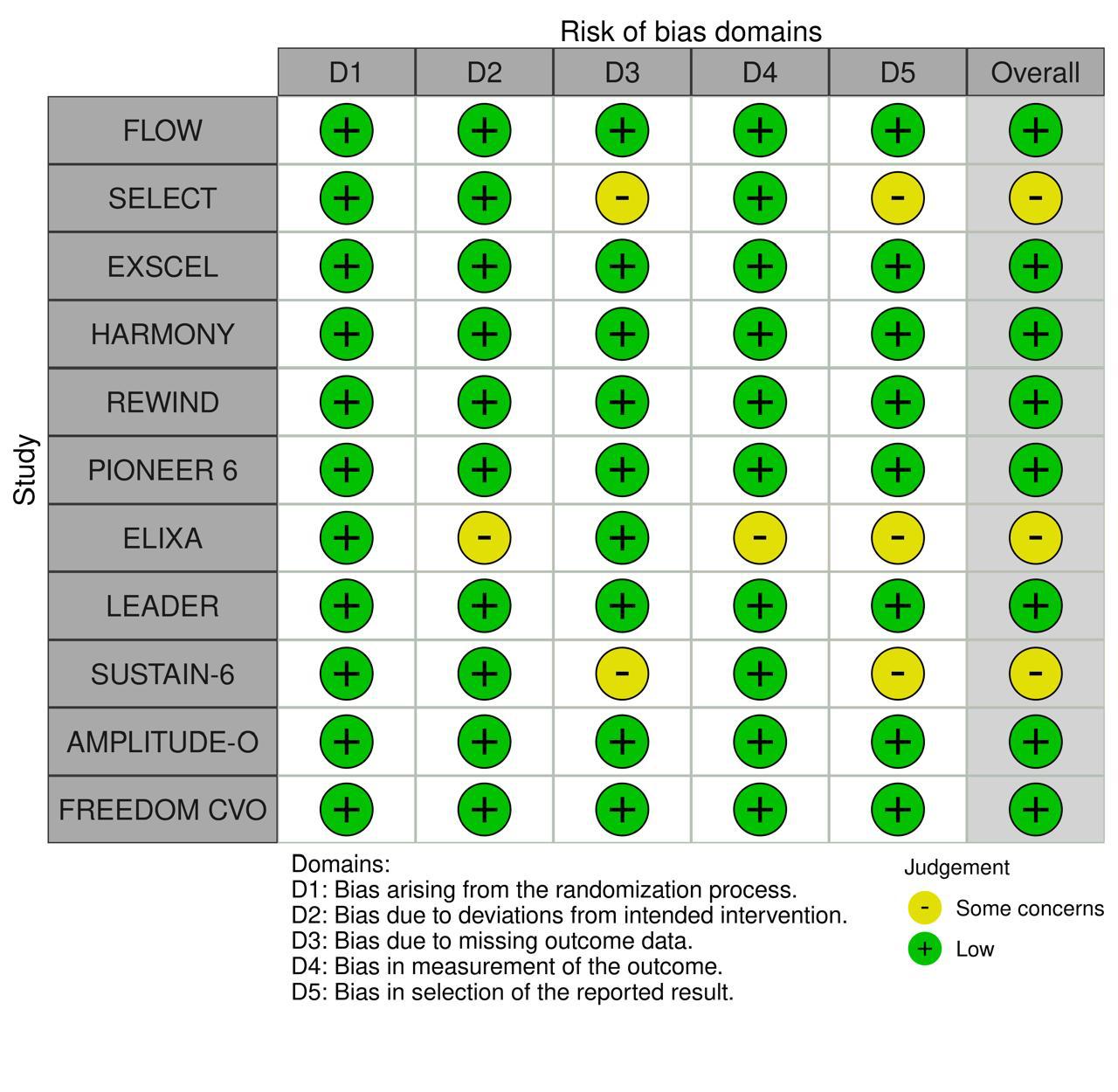


*B) Risk of Bias graph*


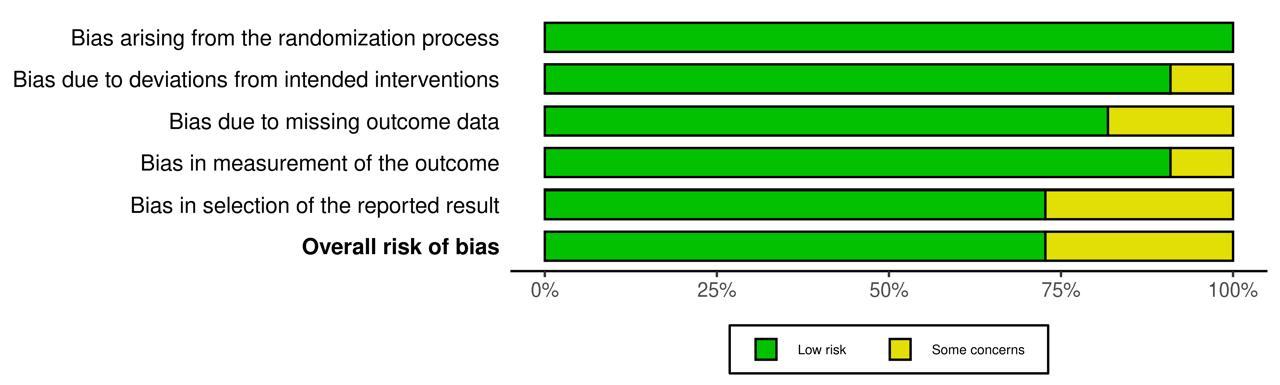


**Supplementary Figure 1:** Quality assessment using Cochrane Risk of Bias tool for randomized controlled trials (RCTs) A) Risk of Bias summary, B) Risk of Bias graph.


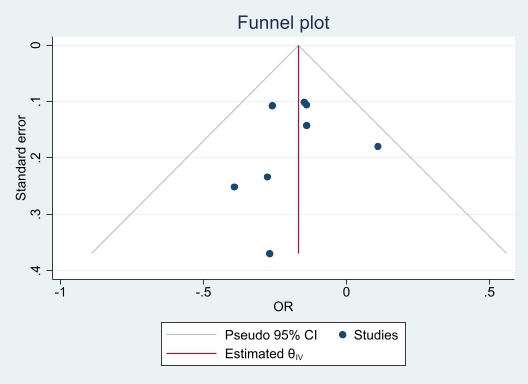


**Supplementary Figure 2:** Funnel plot of primary outcome stroke.


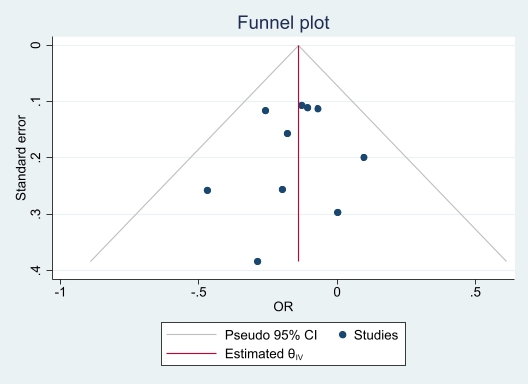


**Supplementary Figure 3:** Funnel plot for non-fatal stroke.


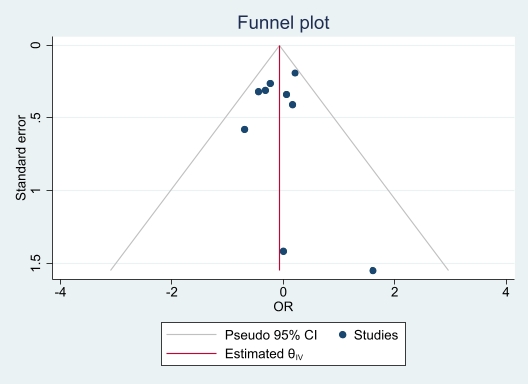


**Supplementary Figure 4:** Funnel plot for fatal stroke.
